# 601 Comparison of Healthcare Resource Utilization in Burns, Frostbite and Necrotizing Fasciitis: A Propensity Score-based Analysis

**DOI:** 10.1093/jbcr/iraf019.230

**Published:** 2025-04-01

**Authors:** Sebastien Normandeau, Justin Gawaziuk, Brenda Comaskey, Lisa Moore, Rae Spiwak, Sarvesh Logsetty

**Affiliations:** University of Manitoba; University of Manitoba; University of Manitoba; University of Manitoba; University of Manitoba; University of Manitoba

## Abstract

**Introduction:**

Patients with burns, frostbite, or necrotizing fasciitis (NF) are recognized to have high rates of healthcare resource utilization (HRU). However, a comparison of HRU between these three groups at a single centre is not well known in the literature. Historically, the HRU of these three groups has not been differentiated with respect to hospital administrative planning. The goal of this study is to compare HRU between these three groups.

**Methods:**

This is a retrospective review of adults acutely admitted for injuries due to burn (n=589), frostbite (n=144) or NF (n=421) at a single Canadian level 1 centre. Two cohorts (burns versus frostbite, burns vs NF) will be created with a ratio of approximately 1:1, propensity-score nearest neighbor matched on age, sex, and geographic location. Descriptive analysis will examine patient characteristics. HRU analysis examines length of hospital stay, number of operations, outcomes (% with autograft, free tissue transfer, in-hospital mortality).

**Results:**

NF patients had significantly mean longer length of stay (32.7d) versus frostbite (24.5d) and burns (18.5d) (p< 0.001). Similarly, a significantly higher proportion of NF patients stayed in ICU (47.2%) versus burns (30.2%) (p< 0.001) as well as free tissue transfer (6.1% vs 3.5% vs 2.4%). Frostbite patients had the highest proportion of amputation (41.7% versus NF with 14.7% and burns with 3.2%)

**Conclusions:**

Generally, NF patients required the highest amount of HRU followed by frostbite and burn patients.

**Applicability of Research to Practice:**

The implications of this study are that burn, frostbite, and NF patients have different rates of HRU, which can directly impact hospital administration resource allocation.

**Funding for the Study:**

N/A

